# Spontaneous ureteral rupture caused by iliac aneurysm: a case report

**DOI:** 10.1186/s40792-018-0555-8

**Published:** 2018-12-20

**Authors:** Hirotaka Sato, Soki Kurumisawa, Tsutomu Saito, Koji Kawahito

**Affiliations:** Department of Cardiovascular Surgery, Jichi Medical University, Yakushiji 3311-1, Shimotsuke, Tochigi 329-0498 Japan

**Keywords:** Ureteral rupture, Spontaneous, Aneurysm

## Abstract

**Background:**

Rupture of the ureter with extravasation resulting from an iliac aneurysm is extremely rare. Herein, we report a case of ureteric rupture with urinary extravasation secondary to an iliac aneurysm.

**Case presentation:**

An 80-year-old man was admitted to our hospital for sudden onset of severe abdominal pain. Contrast-enhanced computed tomography demonstrated a large left internal iliac aneurysm (6.5 cm in diameter) and a ureteric rupture with leakage of contrast media from the left ureter, indicating a spontaneous ureteral rupture. The patient was treated with placement of a ureteral double-J stent under endoscopic and X-ray fluoroscopic guidance and endovascular aortic repair. His postoperative course was uneventful and he was discharged on postoperative day 20. A computed tomography scan at 2 weeks after surgery showed no contrast extravasation from the ureter or end leak.

**Conclusion:**

Combination treatment with ureteral and endovascular stenting is effective in avoiding aneurysmal rupture and the serious consequences of a ureteral rupture, which include a perinephric or retroperitoneal collection, abscess formation, subsequent urosepsis, and graft infection.

## Background

Spontaneous rupture of the ureter is an uncommon condition defined by the non-traumatic extravasation of urine. It is usually secondary to a downstream obstruction by urinary calculi, stricture, or extrinsic compression resulting from malignancies, retroperitoneal fibrosis, and so on [1, 2]. However, an expanding iliac/aortic aneurysm leading to a ureteric obstruction and rupture of the ureter is extremely rare. We herein report a case of ureteric rupture with urinary extravasation secondary to an iliac aneurysm.

## Case presentation

An 80-year-old man was admitted to a nearby hospital for the sudden onset of severe abdominal pain. Plain computed tomography (CT) showed a large left internal iliac aneurysm (IIA). An aneurysmal rupture was suspected, and he was transferred to our hospital for surgical treatment. His past medical history was significant for hypertension and a lack of a history of renal calculus. On physical examination, his blood pressure was 135/75 mmHg; pulse, 90 beats per minute and regular; and temperature, 36.5 °C. His abdomen was distended and tender to palpitation, but was without peritoneal signs. Laboratory data revealed a normal hemogram, with the exception of increased leukocytes (11,400/μL), normal serum electrolytes, blood urea nitrogen level of 20 mmol/L, and serum creatinine level of 1.2 mg/dl. Chest X-ray demonstrated no cardiomegaly or any abnormal findings.

**Fig. 1:**
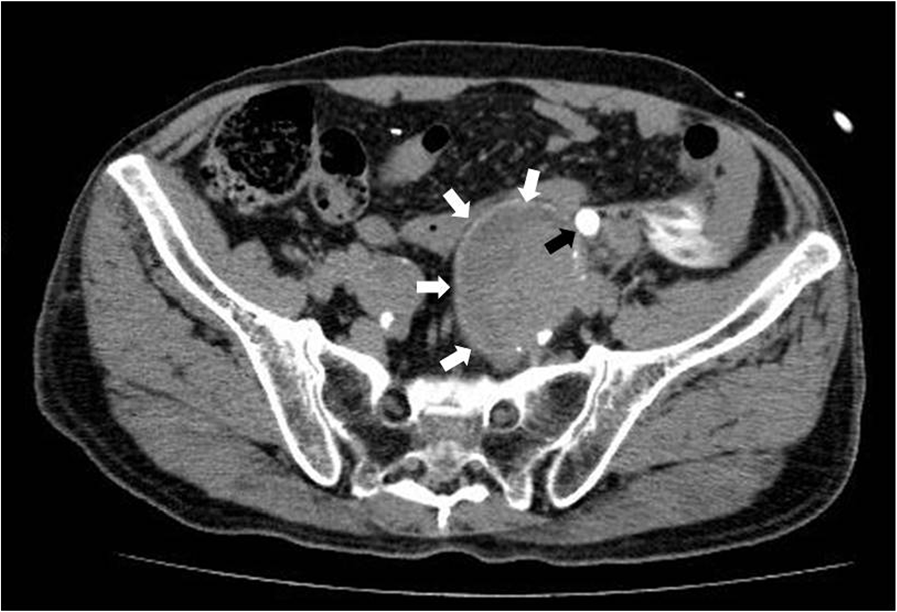
Contrast-enhanced computed tomography (CT) reveals a large left internal iliac aneurysm (white arrows). CT signs of a ruptured aneurysm are not observed. A black arrow indicates the left hydroureter

**Fig. 2:**
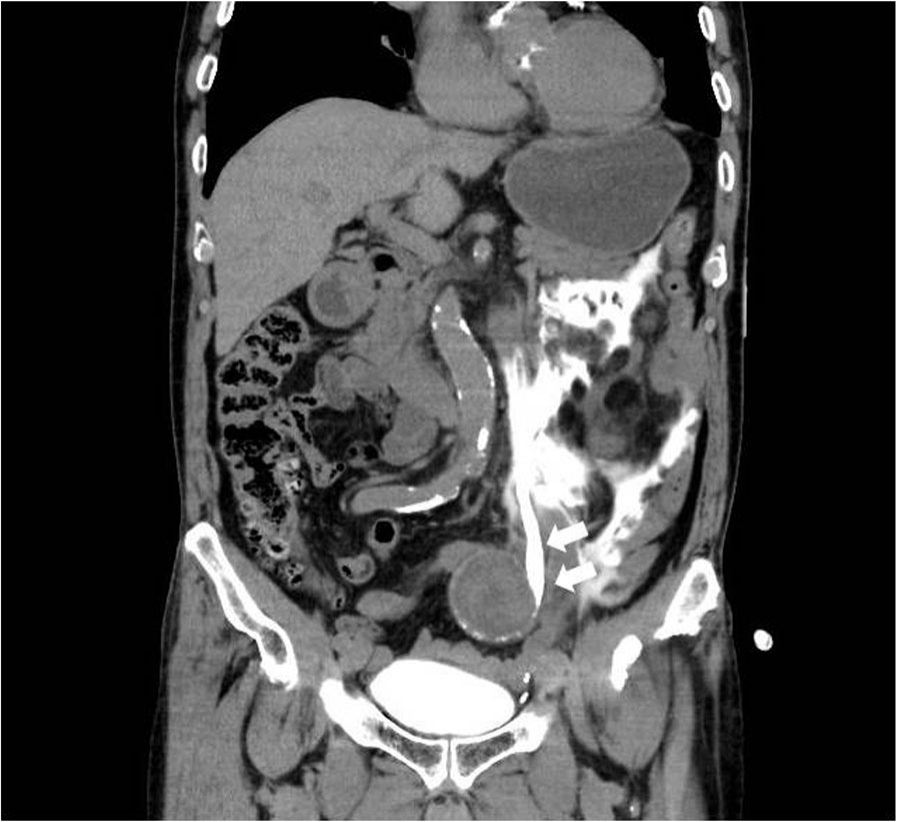
Computed tomography demonstrates a left hydroureteronephrosis and leakage of iodinated urine in the left-side retroperitoneum. Arrows indicate the left ureter compressed by a large iliac aneurysm

Contrast-enhanced CT revealed a large left IIA (6.5 cm in diameter) (Fig. 1). Blood extravasation into the periaortic soft tissue and other CT signs of a ruptured aneurysm were not observed in the arterial phase. Specific CT signs of an inflammatory aneurysm, such as the typical image of soft tissue surrounding the aortic wall enhanced with contrast medium (enhancing periaortic soft tissue, “mantle sign”), were also not observed. However, a left hydroureteronephrosis and leakage of iodinated urine in the left-side retroperitoneum were demonstrated in the delayed phase, indicating a spontaneous ureteral rupture resulting from the direct compression of the ureter by the aneurysm (Fig. 2).

**Fig. 3:**
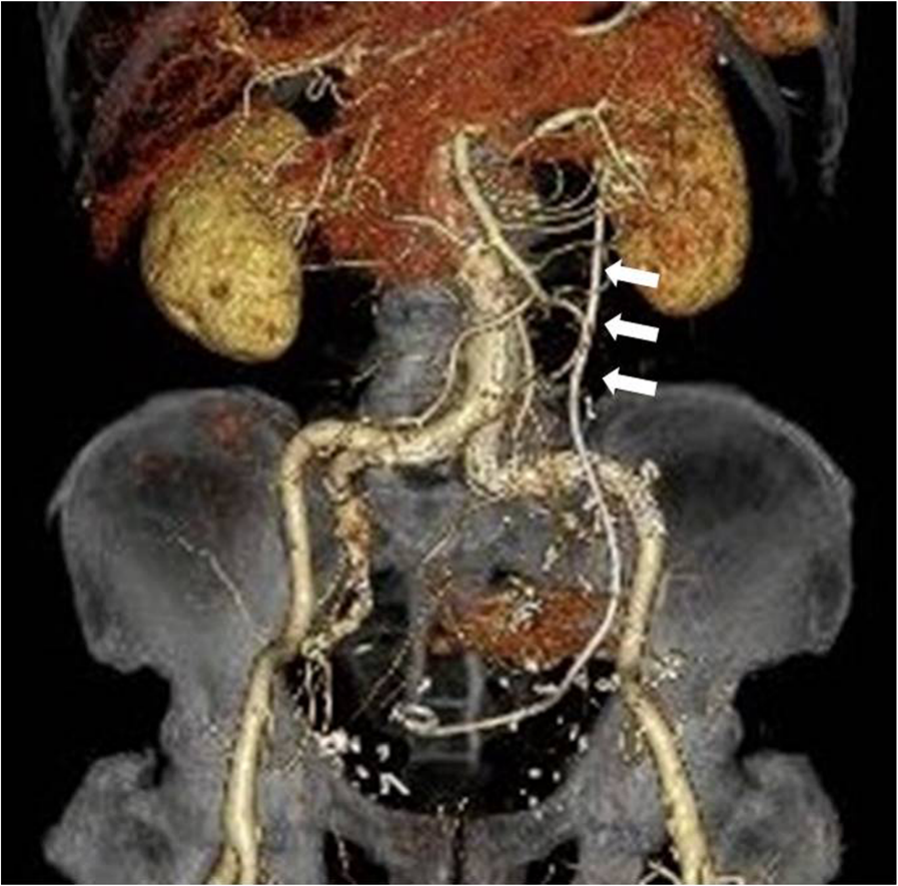
Postoperative computed tomography demonstrates endovascular stent grafting from the left common iliac artery to the left external iliac artery and full occlusion of the internal iliac aneurysm. Arrows indicate the ureteral stent

To avoid the potential risk of graft infection due to urinary extravasation, a ureteral double-J stent was placed under endoscopic and X-ray fluoroscopic guidance. Endovascular aortic repair (EVAR) was performed under general anesthesia on the same day to avoid aneurysmal rupture. The patient underwent endovascular coil deployment within the left IIA and endovascular stent grafting from the left common iliac artery to the left external iliac artery (GORE® EXCLUDER® AAA Endoprosthesis PLC201000J, W. L. Gore and Associates, Inc., Flagstaff, Arizona, USA) (Fig. 3). Final angiography showed full occlusion of the aneurysm and an optimal result.

His postoperative course was uneventful, and he was discharged in good health on postoperative day 20. The progressive reduction of an urinoma was followed by serial CT performed 2 weeks later, which showed its complete resolution.

## Discussion

Spontaneous rupture or extravasation of the ureter due to an iliac/aortic aneurysm is extremely rare; only seven such cases have been reported [3–9]. The majority of ureteral obstruction cases involve inflammatory aneurysms or retroperitoneal fibrosis associated with perianeurysmal fibrosis that ultimately results in the structural compromise of the urinary tract [10, 11]. Similarly, entrapment of the ureter can occur due to the extension of perianeurysmal inflammation into the surrounding tissue in degenerative aneurysms. Serra et al. reported that ureteral obstruction does not occur from the bulging of the aneurysm itself, but rather from the perianeurysmal inflammatory and fibrotic processes [11]. However, with a rapid expansion of an aneurysm with impending rupture, the bulge might induce ureteral extravasation before the compensatory mechanisms of the ureter can function. In our case, the possibility of ureteral occlusion by rapid expansion of iliac aneurysm was not negligible.

Theoretically, the urine spilled into the retroperitoneal space is sterile. Akpinar et al. reported that the initial management of ureteric rupture is usually conservative [1]. However, the potential risk of a retroperitoneal collection, abscess formation, and subsequent urosepsis and graft infection is a concern [12]. Under such circumstances, not only the consequences of an aneurysmal rupture but also the serious consequences of ureteral extravasation should be considered.

Ureteral stenting is useful to avoid the serious consequences of ureteral rupture, including graft infection [9, 12, 13], while the endovascular treatment of aneurysms that lead to a ureteral obstruction remains controversial. Nenezic et al. reported that EVAR has a significant limitation in resolving the compression symptoms of adjacent organs [14]. However, Hechelhammer et al. [15] and Greenberg et al. [16] reported shrinkage of the aneurysmal sac and a significant decrease in periaortic inflammation with EVAR, suggesting that EVAR is appropriate for aneurysms complicated with a ureteral rupture, avoiding a contaminated field and associated retroperitoneal inflammation. Although the precise mechanisms of the regression of the aneurysmal size and periaortic inflammation after EVAR have not been clarified, exclusion of the aneurysm results in an attenuation of the immune response, which may account for the aneurysmal shrinkage and resolution of the perianeurysmal inflammatory process [8].

Although combination treatment was selected to avoid aneurysmal rupture in this case, staged operation might be taken into consideration for unruptured cases to minimize the risk of graft infection as an alternative. The ureteral stenting together with the following antibiotics and surveillance of blood cultures might contribute to decide treatment strategy. Obrand et al. [6] and Edmonds et al. [8] reported the effectiveness of staged approach to avoid graft infection in unruptured cases. Although the strategy should be discussed on a case-by-case basis, the present case was treated successfully by the combined treatment.

## Conclusion

We presented a rare case of ureteral rupture due to the expansion of an iliac aneurysm. Combination therapy, with ureteral stenting and EVAR, is useful in avoiding an aneurysmal rupture and the hostile consequences of a urinoma.

## Abbreviations

CT: Computed tomography
EVAR: Endovascular aortic repair
IIA: Internal iliac aneurysm
